# Post epidemic giardiasis and gastrointestinal symptoms among preschool children in Bergen, Norway. A cross-sectional study

**DOI:** 10.1186/1471-2458-10-163

**Published:** 2010-03-26

**Authors:** Kristin M Mellingen, Anita Midtun, Kurt Hanevik, Geir E Eide, Øystein Søbstad, Nina Langeland

**Affiliations:** 1 Institute of Medicine, University of Bergen, Haukeland University Hospital, N-5021 Bergen, Norway; 2 Centre for Clinical Research, Haukeland University Hospital, N-5021 Bergen, Norway; 3 Research group of Lifestyle Epidemiology, Department of Public Health and Primary Health Care, University of Bergen, N-5020 Bergen, Norway; 4 Department of Health and Social Welfare, City of Bergen, N-5020 Bergen, Norway; 5 Department of Medicine, Haukeland University Hospital, N-5021 Bergen, Norway

## Abstract

**Background:**

A surprisingly low number of children became ill with giardiasis during the large waterborne outbreak of *Giardia lamblia *in Bergen, Norway during autumn 2004. The aim of the present study was to evaluate the prevalence of giardiasis among exposed children one year after an outbreak and compare faecal carriage of *Giardia *and abdominal symptoms among exposed versus unexposed children one year after the epidemic.

**Methods:**

Children between 1 and 6 years old were recruited from the local health care centres in Bergen municipality in the period between June 2005 and January 2006. One faecal sample per child was collected and examined for presence of *Giardia *with a rapid immunoassay antigen test, and parents were asked to answer a questionnaire. A total of 513 children participated, 378 in the group exposed to contaminated water, and 135 in the in the group not exposed.

**Results:**

In the exposed group eleven children had been treated for giardiasis during the epidemic and none in the unexposed group. *Giardia *positive faecal tests were found in six children, all in the exposed group, but the difference between the groups did not reach statistical significance. All six *Giardia *positive children were asymptomatic. No differences were found between the groups regarding demographic data, nausea, vomiting, different odour from stools and eructation. However, the reported scores of abdominal symptoms (diarrhoea, bloating and stomach ache) during the last year were higher in the exposed group than in the unexposed group.

**Conclusions:**

A low prevalence of asymptomatic *Giardia *infection (1.7%) was found among exposed children around one year after the epidemic (1.2% overall prevalence in the study). In the present setting, pre-school children were therefore unlikely to be an important reservoir for continued transmission in the general population.

## Background

*Giardia lamblia *is the most common small intestine parasite found worldwide, causing symptoms such as diarrhoea, abdominal pain, flatulence and malabsorption. However, the disease is uncommon in developed countries. Findings in stool specimens suggest a prevalence in the developing world to be 20-30%, while in the industrialized world it is 2-5% [1].

In November 2004 there was a giardiasis outbreak in Bergen, with almost 1,300 confirmed cases. Based on surplus prescriptions of metronidazole, an estimated total of 2,500 people were treated for symptomatic disease. Among all the reported cases, there were less than 50 children [2]. The total giardiasis incidence was higher than normal for at least half a year after the drinking water had been sanitized (table 1). *Giardia lamblia *has the potential to spread through multiple modes of transmission. A single-source outbreak caused by exposure through a contaminated water source may result in subsequent prolonged propagation through person-to-person transmission in the community [3]. In children the infection is frequently asymptomatic [4-6] and often associated with prolonged carriage of the parasite. One study showed that about half of the children with *Giardia *infection excreted cysts for more than six months [6].

**Table 1:** Frequencies of incident cases of giardiasis in the city of Bergen, Norway 2003-2005

	Jan	Feb	Mar	April	May	June	July	Aug	Sep	Oct	Nov	Dec
2003	2	4	4	0	6	2	0	3	4	4	1	0
2004	2	5	3	3	3	1	0	8	6	22	646	116
2005	35	12	12	15	13	5	9	4	7	8	5	3

Monthly incidence of reported giardiasis in all age groups, Bergen, Norway 2003-2005, source: The Norwegian Surveillance System for Communicable Diseases (MSIS)

A study performed on children in day care centres in Houston found no correlation between the frequency of recent diarrhoeal episodes and the finding of *Giardia*. Stool specimens containing cysts were significantly more frequent in the 13- to 30-month-old children than in children younger than 12 months. The number of enteric symptoms observed in children and the classification of nutritional status based on monthly height and weekly weight measurements did not differ significantly when infected and non-infected children were compared. The study demonstrated that asymptomatic *Giardia *excretion in children younger than 36 months was common and appeared to be well tolerated [6].

The aim of the present study was firstly to compare the prevalence of *Giardia *infection among exposed children one year after a large outbreak. Secondly, an important aim was also to compare children exposed to contaminated water with unexposed children regarding symptoms, demographic data and faecal presence of *Giardia*. Our assumption based on former studies was that children more often than adults have asymptomatic infections, and the hypothesis was that prevalence was higher than what was reflected both during the outbreak and in the incidence the following year. A question raised was if some children could be asymptomatic carriers in the period after the outbreak, and therefore be potential sources of secondary cases in family members and others [2].

## Methods

### Setting

The study was conducted in Bergen, the second largest city in Norway. Bergen had a giardiasis outbreak in November 2004 due to contamination of a drinking water reservoir (Svartediket) supplying some 35,000 of the 275,000 inhabitants of the city (including students not registered as inhabitants). In 2004, there were 15,611 children below five years in Bergen. 1,972 of these received water from Svartediket during the outbreak. Data were collected eight to 17 months after the outbreak. The majority of data (78%) was collected nine to 13 months after the outbreak.

### Subjects

In an a priori calculation it was found that to have (at least) 90% power to detect a difference of 5% vs. 1% prevalence of *Giardia *infection 1 year after the outbreak between the children living in the areas with contaminated water supply and those without at the 5% significance level (at least) 321 children from each area were needed. Altogether 420 and 310 children, respectively, were however contacted and 324 and 189 responded. Moving children to actual contaminated/not contaminated groups using the questionnaire replies resulted in 378 exposed and 135 unexposed of which 356 versus 133 returned faecal samples. A post hoc power calculation using Cytel Studio based on an unconditional exact test for the two proportions gave a power of 85.35%.

There are several health care centres in Bergen. These are municipal institutions that have a key role in following the development for every child aged 0-6 years. Their services are free of charge and are part of the public preventive health services. Children were recruited from three health care centres (Solheimsviken, Engen and Sandviken health centres). The three centres were chosen as they serve a part of the population living close to the city centre, supplied with water from Svartediket and other reservoirs. This way, the children would be demographically similar, except from the water supply, which was the concern of the study. The anticipated exposed group was defined as children with residential address supplied with drinking water from the contaminated reservoir at the time of the outbreak (verified by the supplying list from the City of Bergen, Agency for Water and Sewerage Works). The anticipated unexposed group was defined as children with residential address supplied with drinking water from any reservoir other than Svartediket. Attempts were made to contact parents of all children aged 1-5 years from three health care centres. They were invited to join the study by telephone. Those accepting were contacted by mail.

**Figure 1:**
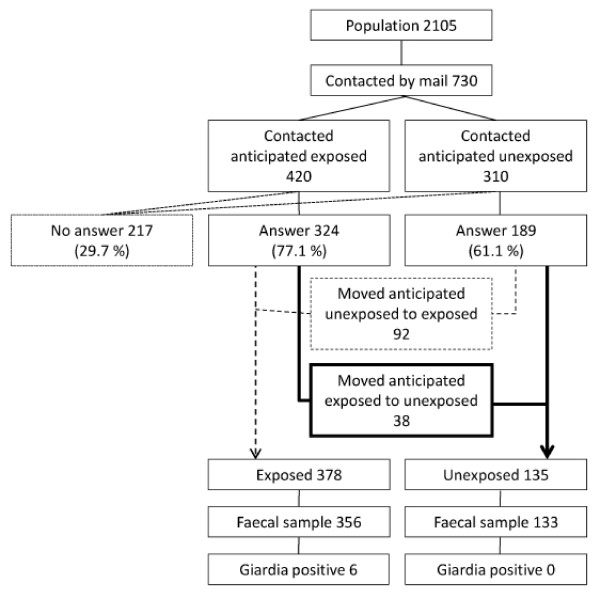
**Participants in the *Giardia *prevalence study among Bergen preschoolers, one year after the 2004 outbreak**. The figure shows how children 1-5 years of age in Bergen were invited to join the study. Children were originally classified based on whether their residential address was supplied by *Giardia*-contaminated water. Those accepting were further classified according to the answers of the questionnaire.

A total of 513 children were included in the study (fig. 1). Ninety two of the children in the anticipated unexposed group had been drinking water from Svartediket at day care centres, at grandparents' etc, based on the answers in the questionnaire. These were transferred to the anticipated exposed group, hence called the exposed group. Children from the anticipated exposed group, whose parents answered they had not been drinking the contaminated water, were moved to the unexposed group. The latter definition of the exposed and unexposed groups seems more correct, and gives more unbiased comparisons between the groups.

Parents were asked for consent. Those accepting the invitation to participate, received written information on the study, questionnaire and equipment for the faecal sample. Parents collected one faecal sample from each child. One reminder was sent to parents who did not return the questionnaire.

### Questionnaire

Parents answered the questionnaire at home. There were three clusters of questions referring to the preceding year: about the child (sex, ethnic background, travelling the last year, treatment for giardiasis, going to day care); about the family (supplied with water from Svartediket, drinking water from Svartediket other places, giardiasis in the family, how many people in the household, siblings in day care); and about the child's health (frequency of gastrointestinal symptoms, and if a doctor was contacted for treatment, did the child lose weight, how much water and milk did the child drink per day).

### Laboratory methods

The faecal samples collected were analyzed using Immunocard STAT! (Meridian Bioscience Inc. Cincinnati, Ohio) antigen test for *Giardia *and *Cryptosporidium*. The test detects antigens in faecal material. The faecal sample was diluted in 10% formalin before analysis.

### Statistics

Data from the questionnaires were recorded in SPSS Data Entry 4.0 (SPSS Inc., Chicago, Illinois). Cross-tables were analyzed using Fisher's exact test and Pearson's chi-square test. For continuous variables, differences between the exposed and unexposed groups were investigated using the Wilcoxon-Mann-Whitney test [7,8]. A significance level of 0.05 was chosen for all tests, and SPSS 15 was used for all analyses.

### Ethics

The study was approved by the Ethics Committee in the Western Health Region and the Ombudsman for Privacy in Research, Norwegian Social Science Data Services. Parents of children who had a positive antigen test (*Giardia*) were contacted via mail. The letter contained information on the parasite, and confirmed that the test had been positive. Parents were encouraged to seek a general practitioner for advice on further investigations and treatment.

## Results

### Demographics

No significant demographic differences were found between the two groups (table 2), except for household size which was larger in the unexposed group (mean = 4.12) than in the exposed group (mean = 3.91). This was statistically significant (p = 0.02).

**Table 2:** Demographic data for exposed and unexposed groups, Bergen, Norway 2005.

	n	Exposed	n	Unexposed	P-value
Sex, n	378		135		0.55^F^
Girls n (%)		181 (47.8)		69 (51.1)	
Boys n (%)		197 (52.2)		66 (48.9)	
Ethnical background, n	331		120		0.21^P^
European n (%)		301 (90.1)		110 (91.7)	
African n (%)		2 (0.6)		3 (2.5)	
Asian n (%)		14 (4.2)		2 (1.7)	
Other n (%)		14 (4.2)		5 (4.2)	
Travelled outside of Northern Europe last year, n (%)	374	144 (38.5)	132	48 (36.4)	0.68 ^F^
Number of members in the household (mean ± SE)	376	3.9 ± 0.1	135	4.1 ± 0.1	0.02 ^W^
Age in months (mean ± SE)	356	36.9 ± 0.9	133	34.9 ± 1.3	0.32 ^W^

F = Fishers exact test (2-sided)

P = Pearson chi-square test (2-sided)

W = Wilcoxon- Mann-Whitney test

SE = Standard error

Demographic data for 513 children aged 0-5 years classified as exposed and unexposed to *Giardia*-contaminated drinking water according to self-report on a mailed questionnaire in the city of Bergen, Norway 2005.

### *Giardia *positive children

Altogether 489 participants submitted a faecal sample for analysis. There were six positive tests, all in the exposed group (Prevalence with 95% Confidence interval 1.23% (0.25%, 2.21%) among the overall study population and 1.69% (0.34%, 3.03%) among the exposed children). Analysis with Fisher's exact test showed this was not significantly different between the exposed and the unexposed (p = 0.20). The six positive children were compared to the rest of the children (table 3). The frequency of travelling was similar in the groups (p = 0.42). The share of children with positive and negative test going to day care, or having siblings going to day care was proportional (p = 0.63 and p = 0.40). There was no significant difference between those with a positive test and those with a negative test regarding symptoms from the gastrointestinal tract (table 3). One of the positive children had a family member who had been treated for giardiasis. None had concurrent *Giardia *and *Cryptosporidium *infection. None of the six positive children had been treated for giardiasis. These analyses were repeated comparing the six positive children to the rest of the exposed group, giving the same results as above (data not shown).

**Table 3:** Characteristics of *Giardia *positive and negative children returning faecal sample, Bergen, Norway 2005

	n	Positive	n	Negative	P-value
Sex, n	6		483		0.22 ^F^
Girls n (%)		1 (16.7)		236 (48.9)	
Boys n (%)		5 (83.3)		247 (51.1)	
Ethnical background, n	4		426		0.21^P^
European n (%)		3 (75.0)		390 (91.6)	
African n (%)		0 (0.0)		5 (1.2)	
Asian n (%)		0 (0.0)		14 (3.3)	
Other n (%)		1 (25.0)		17 (4.0)	
Travelled outside of Northern Europe last year, n	6	1 (16.7)	476	181 (38.0)	0.42 ^F^
Number of people in the household (mean ± SE)	6	4.00 ± 0.26	481	3.99 ± 0.05	0.97 ^W^
Giardiasis in the family, n (%)	6	1 (16.7)	471	54 (11.5)	0.52 ^F^
Child treated for giardiasis, n (%)	6	0 (0.0)	474	10 (2.1)	1.00 ^F^
Going to day care, n (%)	6	4 (66.7)	477	366 (76.7)	0.63 ^F^
Siblings in day care, n (%)	6	3 (50.0)	472	155 (32.8)	0.40 ^F^
Contacted/treated by doctor for gastrointestinal symptoms, n (%)	6	0 (0.0)	464	48 (10.3)	1.00 ^F^
Drinking water, glass (mean ± SE)	6	2.50 ± 0.50	478	2.89 ± 0.07	0.53 ^W^
Drinking milk, glass (mean ± SE)	6	2.33 ± 0.62	478	2.05 ± 0.06	0.60 ^W^
Age at time of testing, months (mean ± SE)	6	34.33 ± 5.15	482	36.38 ± 0.74	0.76 ^W^
Symptoms from the gastrointestinal tract^1 ^(mean ± SE)					
Abdominal pain	6	1.17 ± 0.17	475	1.41 ± 0.03	0.35 ^W^
Nausea	6	1.00 ± 0.00	474	1.18 ± 0.02	0.27 ^W^
Vomiting	6	1.00 ± 0.00	479	1.18 ± 0.02	0.26 ^W^
Diarrhoea	6	1.50 ± 0.34	479	1.57 ± 0.03	0.63 ^W^
Different odour from stools	6	1.17 ± 0.17	471	1.23 ± 0.02	0.83 ^W^
Flatulence	6	1.33 ± 0.21	475	1.65 ± 0.03	0.30 ^W^
Eructation	6	1.33 ± 0.21	474	1.34 ± 0.03	0.94 ^W^

^1 ^values from the questionnaire 1 = seldom/unanswered

2 = sometimes 3 = often

F = Fishers exact test (2-sided)

P = Pearson chi-square test (2-sided)

W = Wilcoxon- Mann-Whitney test

SE = Standard error

Characteristics of 489 children aged 0-5 years returning faecal sample in the City of Bergen, Norway 2005 according to result of a *Giardia *antigen test.

### The exposed group

Children in the exposed group were significantly more troubled with diarrhoea during the last year than those in the unexposed group (p = 0.02). They also had more flatulence (p = 0.03). Except for these symptoms, no differences in symptoms from the gastrointestinal tract were found (table 4). A higher frequency of giardiasis among family members was found in the exposed group compared to the unexposed group (p < 0.001). None in the unexposed group was previously treated for giardiasis. In the exposed group 3% of the children had been treated (p = 0.07). There was not any difference in water intake between the groups. This was true when comparing exposed to unexposed (table 4), and also when comparing *Giardia *positive to the rest of the participants (table 3).

**Table 4:** Symptoms and clinical characteristic for children exposed and unexposed to *Giardia*-contaminated water, Bergen, Norway 2005

	n	Exposed	n	Unexposed	P-value
Giardiasis in the family, n (%)	366	54 (17.3)	135	3 (2.2)	<0.001^F^
Child treated for giardiasis, n (%)	374	11 (3.0)	130	0 (0.0)	0.07 ^F^
Going to day care, n (%)	377	294 (78.4)	132	98 (74.2)	0.34 ^F^
Siblings in day care, n (%)	371	117 (46.1)	131	46 (35.1)	0.45 ^F^
Contacted/treated by doctor for gastrointestinal symptoms, n (%)	360	41 (11.4)	131	9 (6.9)	0.18 ^F^
Drinking water, glass (mean ± SE)	376	2.95 ± 0.08	132	2.83 ± 0.13	0.46 ^W^
Drinking milk, glass (mean ± SE)	375	2.10 ± 0.07	133	1.96 ± 0.10	0.36 ^W^
GI-tract symptoms ^1 ^(mean ± SE)					
Abdominal pain	372	1.42 ± 0.03	132	1.35 ± 0.05	0.32 ^W^
Nausea	370	1.18 ± 0.02	132	1.16 ± 0.04	0.46 ^W^
Vomiting	376	1.19 ± 0.02	132	1.17 ± 0.04	0.36 ^W^
Diarrhoea	377	1.62 ± 0.03	131	1.47 ± 0.05	0.02 ^W^
Different odour from stools	369	1.25 ± 0.03	130	1.17 ± 0.04	0.16 ^W^
Flatulence	372	1.68 ± 0.04	132	1.53 ± 0.06	0.03 ^W^
Eructation	371	1.35 ± 0.03	132	1.30 ± 0.04	0.41 ^W^

^1 ^values from the questionnaire 1 = seldom/unaswered

2 = sometimes 3 = often

F = Fishers exact test (2-sided)

P = Pearson chi-square test(2-sided)

W = Wilcoxon- Mann-Whitney test

SE = Standard error

Symptoms and clinical characteristics for 513 children aged 1-5 years classified as exposed and unexposed to *Giardia*-contaminated drinking water according to self report on a mailed questionnaire in the City of Bergen, Norway 2005.

After excluding the *Giardia *positive children, symptom scores were compared between the two groups. The exposed group still had more diarrhoea (p = 0.01), flatulence (p = 0.02), and more giardiasis among family members (p < 0.001) (data not shown). The frequency of children that had been treated for giardiasis was not significantly different (p = 0.08). Also within the exposed group, the children who had been treated for giardiasis had more abdominal pain than others (p = 0.027).

## Discussion

### Prevalence

*Giardia *prevalence in Denmark, Finland, Norway and Sweden has been estimated to 2.97% in the asymptomatic adult population, and 5.81% in the symptomatic population [9]. In New Zealand, the incidence shows a bimodal pattern, peaking in the 1-4 and 25-44 age groups. The incidence in the youngest age group is nearly double that of the other age groups [10]. A similar pattern has been shown in Vermont [11]. In Bergen, however, statistics from the outbreak showed a peak prevalence for women aged 20-30 years and only 1% of the laboratory confirmed giardiasis cases were children less than 5 years of age [2], giving an estimated prevalence of 0.6%. It must be noted that this prevalence is based only on children seeking medical care for symptomatic disease, and they are not true prevalence data. This estimate has included only children living in the area with contaminated water, and is therefore a maximum estimate. The present data revealed a prevalence of *Giardia *infection 1.2% for the total population of children aged 1-5 years one year after the epidemic. The fact that the prevalence one year after the outbreak is higher than during the outbreak supports the hypothesis that children were asymptomatic, and therefore under diagnosed during the outbreak.

Prevalence of *Giardia *in day care centres are known to be increased compared to the general population. A study from Vermont found a rate of 300 infected per 100,000 (0.3%) in day care and 195 per 100,000 (0.2%) among children not attending day care [11]. In Denver the prevalence among day care attendees were 16% compared to 9% among non attendees [12]. The prevalence in the general public of the Nordic countries is lower compared to these endemic areas [9], but no good estimates on the child population are available. The increased risk may be related to wearing diapers and eating at the day care centre [10]. The present study did not find any difference in frequency of *Giardia *infection between children attending or not attending a day care centre. This suggests that children in day care centres were not at higher risk than the general population during this outbreak. Several factors may have lead to this. Children in day care centres might not drink much water, and therefore be less exposed to primary infection. An epidemiologic study from the outbreak showed increased risk for illness if water intake was in excess of the general public [2]. The authors of that study also points to a study of Norwegian food- and drinking habits that shows children drink less water than adults. Hygienic efforts like hand washing and proper diaper-changing techniques have shown to reduce the spread of pathogens by secondary transmission [12]. The Chief medical officer of infection control emphasized the importance of personal hygiene in limiting the transmission of the parasite in his information to the public. Beyond that, no specific information was given to day care centres during or after the epidemic.

The exposed children experienced more diarrhoea and flatulence than unexposed children. When excluding the *Giardia *positive children the symptom score was unaffected. This may be a sign of undiagnosed giardiasis during the past year, which we could not detect at the time of the study. The *Giardia *positive children, however, had a low symptom score. This is consistent with a range of studies suggesting giardiasis is often asymptomatic in children [1,5].

Previous studies have investigated whether secondary transmission is an important part of the epidemiology of *Giardia*, as asymptomatic children excrete infective cysts in their stools [6]. A longitudinal study of *Giardia lamblia *infection in a day care centre population found that only 22% of infected children had symptoms attributable to *Giardia *infection [5]. Secondary transmission rates from children with giardiasis to household contacts are suggested between 17 and 47% [5,13,14]. One study from New Zealand found housewives and nursing mothers at increased risk of infection, suggesting this is due to person to person transmission from children [15]. In our study this was an important matter of investigation. The aim was to settle whether asymptomatic children may be a relevant reservoir of secondary infections maintaining an increased *Giardia *incidence after the epidemic. The reported incidence of the general population was increased for six months after *Giardia *was eradicated from the water supply. We could not discover any difference between *Giardia *positive and negative children in terms of day care attendance or giardiasis in the family. The present study therefore does not support the theory that asymptomatic children are a reservoir for secondary transmission.

### Findings in the groups

The exposed and unexposed groups were demographically similar except for household size. Children in the exposed group were part of smaller households than the unexposed group (table 2). This is expected, as larger households might move out of the city centre leading to smaller families living in the city centre and thus being more exposed to water from the contaminated reservoir. We did not, however, find any difference in household size when comparing the *Giardia *positive children to the rest of the children (p = 0.791) or to the rest of the exposed group (p = 0.658). The prevalence of *Giardia*, measured by the antigen test, was higher in the exposed group, but not significantly different from the unexposed group. There might be a difference we could not detect due to the small size of the final unexposed group.

### Study limitations

Children in the exposed group scored significantly higher on diarrhoea and flatulence compared to the unexposed group. An important explanation to consider is the recall bias, as parents in the exposed group would possibly worry about, and to a greater extent pay attention to changes in their children's stools. However, if recall bias was the only explanation, one would expect to find similar bias and differences between the groups also in other relevant symptoms than diarrhoea. Due to a similar distribution of other symptoms, we consider this a true difference in the frequency of diarrhoea between the exposed and unexposed children.

Diarrhoea and flatulence may also be indicative for post infectious IBS found in adults after the same outbreak [16]. Our findings may suggest that children in the exposed group had been infected by *Giardia *during the outbreak, and suffer from *Giardia *sequele similar to those seen in adults. However, the questionnaires asked about symptoms for the last year. This period covers a possible *Giardia *infection and also possible persistent symptoms. The study is therefore not designed to consider long term consequences of *Giardia *infection, and further studies are needed on this subject.

In assessing our results, one has to consider the diagnostic test used in the present study. The immunocard STAT! antigen test was chosen because of its good sensitivity (81-93.5%) shown in previous studies [17,18]. A study of the test sensitivity in an adult patient population with symptoms persisting after the infection was also performed in our setting, showing a lower sensitivity of 60% [19]. This may have lead to underestimating of *Giardia *positive children in the present study. However, if the antigen test detected only 60% of the true *Giardia *positive cases in the study, the prevalence would still be low (2%) and similar to the expected prevalence in a Nordic setting.

The classification of exposed and unexposed children is based on parents' answers in a questionnaire. This may lead to a classification bias, but we consider the answer based re-classification to lead to less bias than classification based on residency alone.

## Conclusions

Our study showed a low overall prevalence of *Giardia *in pre-school children, and did not show significantly higher prevalence of *Giardia *in the exposed group compared to the unexposed group. It did show an increased frequency of diarrhoea and flatulence among children who had been drinking *Giardia *infected water. The data does not support previous studies finding high rates of secondary infections related to day care centres attendence. In the present setting, pre-school children were unlikely to be an important reservoir for continued transmission in the general population.

## Competing interests

The authors declare that they have no competing interests.

## Authors' contributions

Data collection was done by KMM and AM. NL supervised the study. GEE supervised the statistical analyses. All authors have participated in the writing and finalization of the paper. All authors have read and approved the final draft submitted.

## Pre-publication history

The pre-publication history for this paper can be accessed here:

http://www.biomedcentral.com/1471-2458/10/163/prepub
